# Acromegaly in Speckle Tracking Echocardiography—A New Cardiac Hypertrophy Phenotype? Case Report and Review

**DOI:** 10.3390/life14111459

**Published:** 2024-11-11

**Authors:** Alexander Suchodolski, Monika Wójcik-Giertuga, Beata Kos-Kudła, Mariola Szulik

**Affiliations:** 1 Doctoral School, Medical University of Silesia in Katowice, 40-055 Katowice, Poland; monikawojcik90@gmail.com; 2 Department of Cardiology and Electrotherapy, Silesian Center for Heart Diseases, Faculty of Medical Sciences in Zabrze, Medical University of Silesia, 40-055 Katowice, Poland; mszulik3@wp.pl; 3 Department of Endocrinology and Neuroendocrine Tumors, Department of Pathophysiology and Endocrinology, Faculty of Medical Sciences in Zabrze, Medical University of Silesia, Ceglana 35, 40-514 Katowice, Poland; endoklin@sum.edu.pl; 4 Collegium Medicum—Faculty of Medicine and Faculty of Applied Sciences, Department of Medical and Health Sciences, WSB University, 41-300 Dąbrowa Górnicza, Poland

**Keywords:** acromegaly, speckle tracking echocardiography, myocardial function, hypertrophy

## Abstract

Background: This study aims to provide a comprehensive overview of speckle tracking echocardiography (STE) findings in patients diagnosed with acromegaly, exploring a potential application for the differential diagnosis of cardiac hypertrophy and guiding clinicians in patient management. To our knowledge, this is the first review showcasing changes in the bull’s-eye pattern in myocardial function after acromegaly treatment, suggesting a possible pattern in this aetiology of left ventricular hypertrophy. Methods: A review of PubMed articles using the search term “speckle tracking echocardiography acromegaly” yielded 11 relevant papers published between 2017 and 2023. The final analysis evaluated each paper’s substantial value and summarised pertinent information. A clinical case of myocardial strain changes before and after pharmacological and neurosurgical treatment is also described. Results: The 11 analysed papers, with patient groups ranging from 19 to 50 individuals, revealed varying results in STE parameters between acromegalic and control groups. Left ventricular strain parameters were commonly assessed, showing discrepancies in different studies. Notably, the MAGYAR-Path Study emphasised left ventricular twist and radial strain abnormalities in acromegaly patients. Furthermore, the interplay between acromegaly and diabetes influenced myocardial function. Drug treatment with somatostatin receptor ligands demonstrated a favourable impact on left ventricular systolic function. The case study we describe in this manuscript showed changes in the posterior basal LV segment, which may be a specific pattern of acromegaly remodelling. Somatostatin therapy and neurosurgery led to the normalisation of global longitudinal strain (GLS) and improvement in myocardial work, as well as improved diastolic function, including enhanced left atrial strain (LAS) as well as a visible elastic recoil sign (ERS). Conclusions: While the available literature on STE in acromegaly is limited, our analysis suggests potential applications in differentiating hypertrophy aetiologies and monitoring cardiac function post-treatment. The results underscore the need for more interdisciplinary research to optimise patient management. The bull’s-eye pattern with posterior basal segment strain impairment we describe may be used to differentiate this entity.

## 1. Introduction

Acromegaly is a rare endocrine disease diagnosed with a frequency of up to 40–70 cases per million people [1,2,3]. Due to its scarce occurrence and uncharacteristic first symptoms, a significant aspect of this condition is that it takes a relatively long time to diagnose, with the diagnosis process often lasting up to 7–10 years [2,3]. This means that such patients encounter many other specialists before being diagnosed by an endocrinologist. Physicians’ awareness of this disease and its systemic manifestations seems to be crucial in accelerating the right diagnosis and, therefore, the initiation of an effective treatment regimen. Acromegaly is most often caused by a pituitary adenoma, which is related to an excessive production of growth hormone (GH) and, consequently, an increase in the concentration of insulin-like growth factor 1 (IGF-1) [3,4]. Somatotropic adenomas sporadically occur in most cases; less often, they may be genetic; and they can be diagnosed in multiple endocrine tumour syndrome multiple endocrine neoplasia (MEN) type 1 and type 4, familial isolated pituitary adenoma (FIPA), pituitary adenoma with paraganglioma/pheochromocytoma (3Pas), or with X-linked acrogigantism [5]. Less often, acromegaly may also be associated with the ectopic secretion of somatoliberin (GHRH, growth hormone-releasing hormone) or GH by NENs (NENs, neuroendocrine neoplasms), especially with the localisation of the primary tumour in the thymus, bronchi, or pancreas [5].

For the diagnosis of acromegaly, the presence of clinical phenotypic symptoms is significant [5]. Typical are mainly changes in facial appearance such as a thickening of the facial features, swelling of soft tissues, enlargement of the facial bones (especially the mandible and nose) and tongue, enlargement of the hands and feet and internal organs (visceromegaly) [5]. The symptoms of acromegaly may affect, among others, the circulatory system (dyspnoea, swelling of the lower limbs associated with heart failure, arterial hypertension, or cardiac dysrhythmia), the respiratory system (obstructive sleep apnoea, snoring, or impaired patency of the upper respiratory tract), and metabolic complications (diabetes mellitus or impaired glucose tolerance) [5].

If acromegaly is suspected, IGF-1 levels should be screened [5]. When the IGF-1 concentration is increased (relative to age and gender), performing a GH secretion inhibition test after oral administration of 75 g of glucose (OGTT, oral glucose tolerance test) is recommended. A lack of inhibition of GH secretion in the OGTT below 1.0 μg/L (ng/mL) or below 0.4 μg/L in the case of ultrasensitive GH tests indicates active acromegaly [5]. In the case of existing diabetes, instead of OGTT, GH concentration should be determined several times, e.g., every 30 min for 2–3 h [5]. Basic imaging diagnostics include magnetic resonance imaging (MRI) of the pituitary gland with contrast. If no pituitary tumour is imaged, the diagnostics will be expanded to include appropriate tests for diagnosing neuroendocrine neoplasms (NENs) [5].

The main cause of death in acromegaly is, among others, cardiovascular disease [6]. For instance, left ventricular hypertrophy, defined as an increase in left ventricular mass of more than 150 g (>90 g/m^2^) for women and more than 200 g (>103 g/m^2^) for men, can occur in the course of this disease. Additionally, a thickening of the septal and posterior wall exceeding 9 mm in women and 10 mm in men should be measured using imaging techniques, most commonly echocardiography, due to its low cost and, therefore, availability, allowing for serial measurements. The development of acromegalic hypertrophic cardiomyopathy can often be asymptomatic [6]. This makes proper cardiovascular imaging even more crucial, as it allows for the identification of patients with such changes in the myocardial structure at an early stage, when interventions are more effective and the observed abnormality could resolve after proper treatment [7].

Such conditions are often diagnosed without imaging when patients have severe complications resulting in symptoms. According to some data, cardiovascular diseases may cause death in up to 23–50% of cases of acromegaly patients [8,9].

Because of this, it is recommended that, at the time of diagnosis, patients be examined by a cardiologist to discover cardiovascular complications and so that a suitable follow-up can be planned [5]. According to the guidelines, electrocardiogram and echocardiographic examinations should be performed yearly, and blood pressure should be measured during every examination in patients diagnosed with acromegaly [5]. The awareness of cardiac hypertrophy among cardiologists as one of the clinical manifestations of acromegaly allows for early diagnosis and referral to appropriate centres for further treatment, which increases the effectiveness of treatment and significantly improves the survival rate.

Classic echocardiographic findings in patients with this condition predominantly include left ventricular hypertrophy and various valvular pathologies, with mitral regurgitation being the most frequently observed. A recent single-centre cohort study provided substantial data on the prevalence of these valvular abnormalities, reporting that valvular pathologies were present in an impressive 87.3% of patients with this condition. Of these, significant lesions were identified in 14.6% of cases, highlighting the clinical relevance of these findings [10].

In addition, an autopsy study conducted on patients with acromegaly revealed that myocardial hypertrophy was present in 93% of the cases examined, emphasising the widespread nature of this cardiac abnormality in the population. Notably, the same study also found that cardiomegaly, or an enlarged heart, was present in 73% of the cases among patients who did not have a prior diagnosis of hypertension. This finding is particularly significant, as it suggests that the hypertrophic changes observed were not merely secondary to hypertension but likely a direct manifestation of the underlying condition [11].

Two-dimensional speckle tracking echocardiography (2D-STE) has emerged as a transformative tool in the nuanced assessment of myocardial contractility, offering unprecedented sensitivity and precision. This advanced imaging modality is particularly valuable in evaluating patients who present with preserved ejection fraction (EF), a cohort in which traditional echocardiographic parameters may not fully capture subtle myocardial dysfunction. The application of 2D-STE allows for the detection of early myocardial abnormalities that might otherwise go unnoticed, enabling clinicians to initiate targeted therapeutic interventions promptly.

In the context of this patient population, the ability to identify early signs of subclinical myocardial impairment is crucial. Myocardial strain, as measured by 2D-STE, provides a quantitative assessment of myocardial deformation, reflecting the underlying contractile function of the myocardium with high fidelity. This is especially pertinent given that patients with preserved EF can still harbour significant myocardial dysfunction that may not be apparent through conventional echocardiographic measures such as left ventricular ejection fraction (LVEF) alone.

The clinical implications of early detection through 2D-STE are profound. By identifying subtle abnormalities in myocardial contractility before they manifest as overt heart failure or other severe cardiac complications, clinicians can tailor therapeutic strategies to the individual patient’s needs. This pre-emptive approach not only mitigates the progression of myocardial disease but also has the potential to improve long-term outcomes by preserving cardiac function and preventing irreversible damage [6].

Moreover, the use of 2D-STE in routine clinical practice underscores the evolution of echocardiography from a primarily diagnostic tool to one that plays a pivotal role in the ongoing management and monitoring of cardiac patients. As we continue to refine our understanding of myocardial mechanics through advanced imaging techniques like 2D-STE, the ability to deliver personalised, evidence-based care will only continue to improve. This ensures that interventions are both timely and effective, thereby reducing the risk of permanent cardiac complications and enhancing the quality of life for patients with preserved EF. Speckle tracking echocardiography (STE) is a novel imaging method that allows the tracking of small fragments of the myocardial wall called “speckles” using computer analysis. This technique provides more precise myocardial function data than traditional methods like ventricular ejection fraction (EF) calculation. Therefore, changes in systolic function can be detected earlier. Echocardiographic images, which, using conventional methods, seem similar, can be distinguished. One of the fields where this is used is imaging differential diagnosis of left ventricular myocardial hypertrophy, like in the case of acromegaly patients [12]. As a recent study shows, it is precise enough to even differentiate different genetic subtypes of hypertrophic cardiomyopathy (HCM) [13]. Moreover, this technique is more cost-effective than cardiac magnetic resonance, which is usually used in this clinical scenario, allowing for repeated testing and follow-up [14].

Surprisingly, a thorough review of the existing literature revealed a distinct lack of studies specifically addressing the differentiation of myocardial hypertrophy due to acromegaly from other aetiologies using speckle-tracking echocardiography (STE). This is particularly striking given the extensive body of research that has focused on utilising STE to characterise myocardial hypertrophy in other conditions, such as hypertension, hypertrophic cardiomyopathy, and infiltrative diseases like amyloidosis. These conditions have long been recognised as important causes of ventricular hypertrophy, and as a result, they have garnered considerable attention within the STE research community, leading to the accumulation of a substantial amount of data.

In contrast, despite the well-documented cardiovascular manifestations of acromegaly, including its association with cardiomyopathy and hypertrophic changes, there remains a significant gap in the literature regarding the application of STE to this specific patient population. The absence of studies in this area is noteworthy, especially when considering the unique pathophysiological mechanisms underlying acromegalic cardiomyopathy, which may distinguish it from other forms of hypertrophy and warrant targeted investigation through advanced imaging modalities like STE.

This paper seeks to address this gap by providing a comprehensive summary of the current literature on speckle-tracking echocardiography findings in patients diagnosed with acromegaly. By collating and analysing the available data, we aim to offer clinicians valuable insights that could assist in differentiating the aetiology of cardiac hypertrophy in this complex patient population. Furthermore, it is our hope that this review will serve as a catalyst for future research, encouraging imaging specialists to explore and identify potential characteristic patterns of STE specific to acromegalic cardiomyopathy.

Such advancements could mirror the progress already achieved in the study of other hypertrophic conditions, where distinct STE patterns have been established, enhancing diagnostic accuracy and informing treatment decisions. Ultimately, the identification of STE features unique to acromegaly could significantly improve the ability of clinicians to distinguish this condition from other causes of hypertrophy, thereby facilitating more personalised and effective management strategies for affected patients.

## 2. Materials and Methods

The available medical literature was reviewed with the intention to find and analyse all papers concerning echocardiographic speckle tracking features of patients with diagnosed acromegaly. To do this, we evaluated all English-language original articles found on PubMed using the search term “speckle tracking echocardiography acromegaly”. We found 12 original articles published between 2017 and 2023. One paper centred around the topic of the mitral valve annulus and was excluded, as this topic was beyond the scope of this review [15]. The final analysis included 11 papers. The authors evaluated each paper’s substantial value, and the information is summarised in this manuscript.

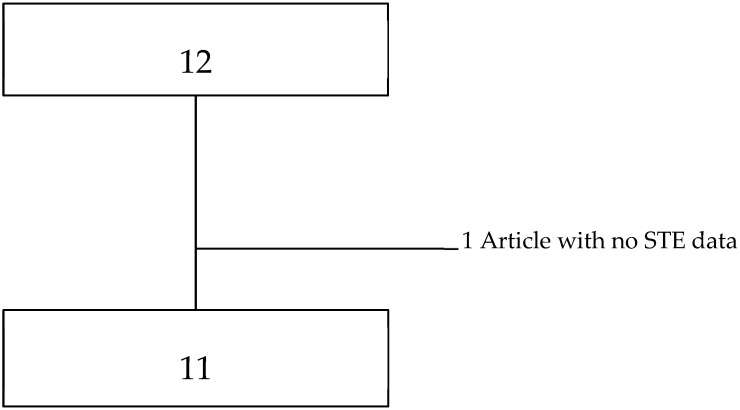



## 3. Results

We evaluated 11 papers. All of them included a small patient group. The number of acromegalic patients in these papers ranged from 19 to 50. Most of the papers had healthy control groups (9/11). Differences between the two groups in terms of STE parameters were found in nine out of eleven studies. In only two manuscripts, no significant differences were detected [16,17]. Left ventricular strain parameters were assessed in most of the evaluated studies (9/11). Left atrial strain was analysed in three papers and right atrial strain in one paper. Notably, only this parameter seems to increase in patients with acromegaly compared with healthy controls [18]. No papers concerning right ventricular strain in this patient group could be found. In the first paper regarding this topic from the year 2017, by Volschan et al., patients with acromegaly and no detectable heart disease according to conventional echocardiographic protocol and a healthy control group were compared [17]. While left ventricular mass index (LVMi) increased in the study group, there were no significant differences in global longitudinal strain (GLS) between the two groups. However, a negative correlation between LVMi and GLS could be found. In the study, the absence of significant differences may be attributed to the fact that the majority of patients had previously undergone treatment for acromegaly, with only 37.8% classified as treatment-naïve. As demonstrated in our subsequent case report, appropriate therapeutic interventions can lead to the normalisation of myocardial strain and diastolic echocardiographic parameters [17]. In the study with the largest population (n = 50), a significant decrease in left ventricular and left atrial strain was observed. Left ventricular mass index (LVMI) was an independent determinant of reduced global longitudinal LV strain [19]. The same negative correlation was shown in a study by Popielarz-Grygalewicz et al. [20]. This could mean that the metabolic effects of acromegaly do not cause a decrease in myocardial function but are a downstream effect of the wall thickening caused by it. Another finding supports this assumption: this reduction correlates with longer disease duration. In a recent study by Popielarz-Grygalewicz et al., a comparison between acromegalic patients with a 10-year vs. 5-year disease duration showed a statistically significant difference in terms of GLS [20]. In contrast, a study by Gadelha et al. showed no significant differences in strain parameters between patients with acromegaly and healthy controls, despite an estimated disease duration of 11 years. However, this estimation relied on patient anamnesis and an analysis of historical photographs, which may be inaccurate.

The authors compare their results to the previously mentioned paper by Volschan et al. Similarly, as in the latter paper, not all patients were examined during active disease periods. It was reported that 72% of the study population exhibited uncontrolled disease. This may explain why significant differences in STE parameters were not found. The authors of the study reported, however, differences in terms of global radial strain, but they concluded that this finding is of no significance as this type of strain can be influenced by many factors, making it a less reliable parameter. Moreover, while there are differences, the values in both groups where within the reference values [16].

Six articles were part of the MAGYAR-Path Study (Motion Analysis of the Heart and Great Vessels by three-dimensional speckle-tracking echocardiography in pathological cases). Speckle tracking echocardiography provides more information than just longitudinal analysis of wall motion and, therefore, myocardial function. Among other parameters that can be assessed is left ventricular twist. In a study by Kormanyos et al., this parameter was impaired in patients with acromegaly [21]. However, in another paper, no differences were found compared to healthy controls [16]. The manuscripts did not explain these differing findings. However, there is the possibility that a confounding factor was not considered and measured. Another parameter, radial strain, was significantly impaired compared to the control group in two of the analysed studies [16,22]. Another study showed no differences in this measurement when acromegalic patients with and without diabetes were compared. These findings suggest that acromegaly appears to be a primary contributor to the observed impairment, as diabetes demonstrated no significant effect in a comparative analysis [23]. The interplay between two different endocrinologic disorders, namely diabetes mellitus and acromegaly, was further investigated in a study by Nemes et al., which showed more differences in myocardial function between acromegalic patients with and without diabetes. They found that acromegalic patients without diabetes had increased global and mean segmental LV (left ventricular) radial strain, while patients with both diseases had results comparable with the healthy control group [23]. This difference could not be detected using longitudinal strain. The authors do not provide an explanation for these findings; however, studies of this nature indicate the need for more detailed investigations to draw robust conclusions regarding cardiac mechanics in patients with acromegaly, particularly given that comorbidities such as diabetes may significantly influence the measured echocardiographic parameters [20]. Acknowledging these findings may aid in selecting optimal medical therapy for such patients. Given that sodium-glucose cotransporter-2 inhibitors (SGLT2i) have demonstrated significant benefits in the treatment of heart failure, including cases with preserved ejection fraction, particularly in patients with concurrent diabetes, they may emerge as a recommended treatment option. However, further research is warranted to substantiate this approach [24].

LA (left atrial) strain is a novel method of assessing the function of this part of the heart, and it is also an important marker of diastolic dysfunction, which seems to be a problem in this patient population. All three studies where this parameter was analysed showed impairment in patients with acromegaly [19,21,25]. Uziębło-Życzkowska et al. reported, for instance, an impairment across all left atrial strain parameters. The authors even excluded diabetic patients to determine whether these differences remained detectable, which they did. This finding aligns with observations in the patient presented in our case report, in whom significant improvement in these echocardiographic measures was observed following surgical and pharmacological treatment for acromegaly without the use of any cardiac medications [25]. In the only study regarding right atrial (RA) strain in this patient population so far, the researchers found that despite volumetric parameters being increased compared to healthy controls, RA strain—and, therefore, RA function—increased. This effect was pronounced in active acromegaly [18].

The treatment of acromegaly seems to have a beneficial effect on heart function. In a recent study, preoperative somatostatin receptor ligand (SRL) treatment showed a favourable effect on left ventricular systolic function (measured using GLS), which could be measured after 3 months of drug treatment [6]. We summarise the mentioned papers in Table 1.

## 4. Case Report

**Figure 1:**
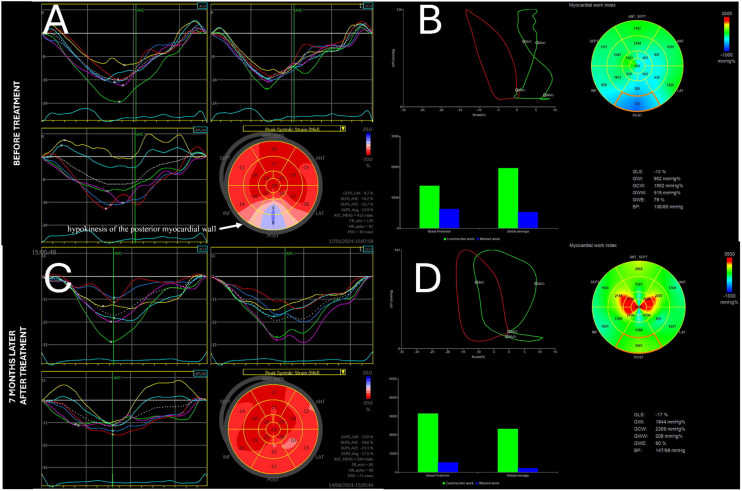
Echocardiographic evaluation of systolic function. (**A**)—GLS with hypokinesis of posterior myocardial wall. (**B**)—Myocardial work, dysfunction also visible in basal segment of posterior wall. (**C**)—GLS after treatment. Visible improvement. (**D**)—Improved myocardial work after treatment.

**Figure 2:**
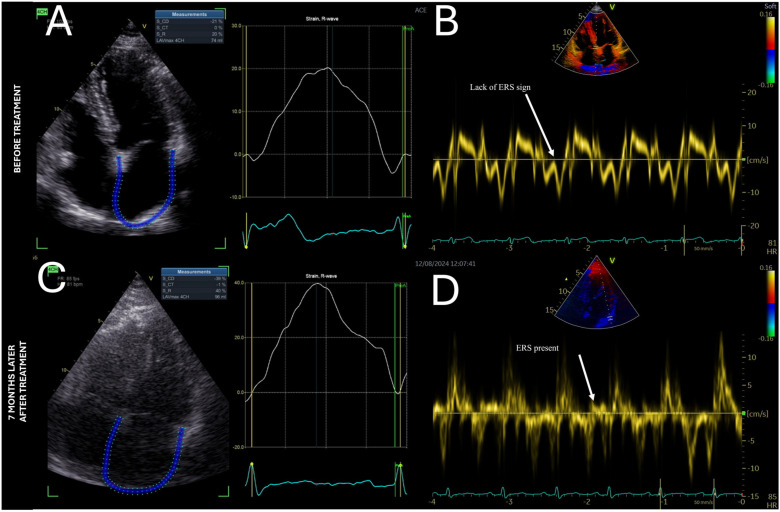
Echocardiographic imaging showing improvement in diastolic function after treatment. (**A**) Speckle tracking of the left atrial walls showed a peak strain rate of 20%, which proves second-degree diastolic dysfunction. (**B**) Tissue Doppler imaging of the IVS shows no ERS, indicating diastolic dysfunction. (**C**) After treatment, LA strain increased significantly to 40%. (**D**) ERS starts to be visible on the lateral wall.

A 47-year-old male patient presented with typical clinical features of acromegaly, such as tongue enlargement, hands, feet, and thickened facial features. High levels of insulin-like growth factor-1 (IGF-I), at 1591.00 ng/mL (84.6–211), and growth hormone (GH), at 8.720 ng/mL (0.030–2.470), and no suppression of GH levels <1.0 ng/mL in the oral glucose tolerance test (OGTT) after the administration of 75 g glucose confirmed the diagnosis of acromegaly. The patient underwent a routine cardiologic check-up. Echocardiography showed a mildly thickened interventricular septum (IVS) of 12 mm. LVEF was preserved (54%). Speckle tracking analysis showed a reduced GLS of −13.9% and hypokinesis of the posterior myocardial wall (Figure 1A). While E/e’ was normal, we noticed no elastic recoil sign (ERS) in the IVS—a novel diagnostic sign of diastolic dysfunction [27] (Figure 2B). The posterior basal segment was dysfunctional in myocardial work analysis. The patient’s Global Work Index (GWI) value was 902 mmHg%, Global Constructive Work (GCW) was 1952 mmHg%, Global Wasted Work (GWW) was 519 mmHg%, and Global Work Efficiency (GWE) was 79% (Figure 1D). Speckle tracking of the left atrial walls showed a peak strain rate of 20%, which proves second-degree diastolic dysfunction [28] (Figure 2A).

The patient underwent surgical removal of a pituitary adenoma (MR images shown in Supplementary Materials). A significant decrease in insulin-like growth factor-1 (IGF-I) level was observed, at 833 ng/mL (84.6–211 ng/mL), as well as growth hormone (GH) normalisation to 1.58 ng/mL (0.030–2.470 ng/mL). Speckle tracking analysis during follow-up 7 months later showed a normal GLS of −17.5%. The previously observed wall motion abnormality was no longer present (Figure 1C). This shows that acromegaly treatment not only relieves the classical symptoms of this disease but also can be beneficial in terms of myocardial function. The elastic recoil sign (ERS), which was previously absent, could now be seen (Figure 2D), which could indicate enhanced diastolic function of the left ventricle. Another indicator of this was a significant improvement in LA strain to 40% (Figure 2C). Moreover, improvement in terms of myocardial work was also measured: GWI, 1844 mmHg%; GCW, 2309 mmHg%; GWW, 208 mmHg%; and GWE, 90% (Figure 1D).

It is noteworthy that this was achieved without cardiac-specific pharmacological therapy.

## 5. Discussion

Cardiomyocytes express receptors for both GH and IGF-1 [29]. In the heart, GH can act directly on peripheral tissues or through IGF-1, produced in the liver [29]. Most often, acromegaly is caused by a pituitary adenoma; however, it may also be associated with ectopic secretion of somatoliberin (GHRH, growth hormone-releasing hormone) or GH by neuroendocrine neoplasms (NENs, neuroendocrine neoplasms), especially with the localization of the primary tumour in the thymus, bronchi, or pancreas [5]. IGF-1 mediates the effects of GH and causes many autocrine and paracrine effects [4]. Although GH, due to the possibility of releasing nitric oxide (NO), affects the vessels, maintaining appropriate peripheral resistance and vascular reactivity, and although it is also used for therapeutic purposes, an excessive and long-term secretion of GH has an unfavourable effect [30]. Acromegaly, among various endocrine diseases, may lead to the development of hypertrophic cardiomyopathy and heart failure, both acute and chronic, which should be taken into account in the differential diagnosis of these diseases [31]. GH/IGF-1 leads to biventricular concentric cardiac hypertrophy, mainly left ventricular hypertrophy, including initially diastolic and then systolic heart dysfunction [29]. Diastolic impairment may remain asymptomatic for several years, regardless of the estimated duration of the disease [32]. It is still to be determined whether torsion, twist, and untwist changes precede diastolic dysfunction. Systolic function measured by ejection fraction (EF) in echocardiography is often normal, but only in the late stages of the disease can it be changed [6]. GLS allows for the detection of subtle changes in systolic function in earlier stages before the ejection fraction deteriorates. A further development of this parameter is myocardial work, which incorporates afterload into analysis through simple hand-cuff measurements of blood pressure. This allows for results independently of loading conditions, unlike LVEF and GLS. This technique is increasingly used in clinical practice, including the differential diagnosis of left ventricular hypertrophy [33,34].

Diastolic heart dysfunction develops in acromegaly, even when the disease has not yet been complicated by coronary artery disease, hypertension, or diabetes [30]. Atrial strain is a promising diagnostic utility for the assessment of diastolic dysfunction, which precedes further pathological findings. It has proven to be a sensitive marker, even differentiating aetiologies in HCM and cardiac amyloidosis patients [35].

Significantly increased GH and IGF-1 concentrations cause sodium retention and increased extracellular volume, including increased blood pressure [9]. The development of end-stage heart failure may resemble dilated cardiomyopathy in many respects [30], or even restrictive cardiomyopathy, which is the final stage of hypertrophy and diastolic and then systolic dysfunction. In long-term acromegaly, the heart can be enlarged, its chambers dilate, and ventricular relaxation is impaired [30]. According to some data, patients show a significant degree of cardiac fibrosis and even an eight-fold increase in the content of connective tissue in an endomyocardial biopsy, which strongly correlates with the duration of the disease [30].

Additionally, patients with acromegaly are characterised by hyperinsulinemia and insulin resistance, and an appropriate sensitivity of the heart muscle to insulin is necessary to maintain the proper function of the circulatory system. GH and IGF-1 may also stimulate some pathways, such as the phosphatidylinositol 3-kinase (PI3K/AKT) pathway and/or the mitogen-activated protein kinase (MAPK) pathway, associated with cardiac hypertrophy [29,36,37]. Wolf et al. examined 26 patients with active acromegaly before and after treatment and 31 controls using cardiac MRI in a prospective cross-sectional study. According to these authors, acromegaly causes a disease-specific form of hypertrophic LV remodelling, characterised by increased extra- and intracellular mass [38].

The primary goal of acromegaly treatment is to normalise GH and IGF-1 secretion and to remove or significantly reduce the mass of the pituitary tumour [5]. In the case of effective treatment of acromegaly, including surgery for pituitary adenoma and normalisation of GH and IGF-1 levels, myocardial function may normalise even in the case of severe fibrosis [39]. The use of treatment with long-acting SAA may lead to improvements in systolic function and cardiac fibrosis [40]. Wolf et al. reported that treatment of acromegaly can reduce the LV mass index and intracellular mass [38].

This makes accurate clinical diagnosis crucial, as effective treatment options are available. According to the guidelines in acromegaly patients with no known cardiovascular disease, echocardiography could be performed if cardiomyopathy is diagnosed and further management is indicated [5,41]. Cardiologists, especially cardiology imaging specialists, encounter cardiac hypertrophy often. According to the Framingham study, echocardiographic criteria for left ventricular hypertrophy were fulfilled in 15.5% of men and 21.0% of women. Notably, in multivariate analyses, left ventricular mass was significantly associated with cardiovascular and all-cause mortality [42]. Considering this, cardiologists should remember that acromegaly can be a potential explanation of this finding and know the clinical characteristics of this disease to guide further diagnostics of these patients.

Acromegaly seems to present as a separate hypertrophic myopathy in terms of STE imaging. In other conditions presenting with hypertrophy of the left ventricular wall, a decrease in GLS is observed, with differences in quantity and regional distribution. In patients with acromegaly, most of the evaluated studies showed a similar decrease in strain parameters. Acromegaly-specific patterns with emphasis on regional variations could be helpful in echocardiographic differential diagnosis, as already established in other myocardial hypertrophies [11]. In the case described in Figure 1, posterior wall strain dysfunction is diagnosed. Another use of the findings discussed in this paper could be patient follow-up. STE-derived parameters are robust, repeatable, and, most importantly, more sensitive markers of systolic function than LVEF. This allows for the detection of subclinical differences. This makes this parameter suitable for monitoring cardiac function—especially after acromegaly treatment. This approach has even been adopted in the context of chemotherapy in the recently published cardio-oncology guidelines [43]. Research shows successful therapy may also improve these patients’ heart function, as shown in research using traditional and speckle tracking echocardiography parameters [6,44].

## 6. Conclusions

The current guidelines on the cardiovascular imaging of patients with hypertrophic cardiomyopathy do not mention this group of patients as a phenocopy of HCM [45,46]. The literature regarding speckle echocardiography findings in this patient population is scarce and limited to the publications listed above, although cardiac hypertrophy in acromegaly is a textbook sign of this disease. The endocrinologic guidelines on diagnosing and treating acromegaly also do not emphasise the possible role of STE [47]. More interdisciplinary research in this area is needed to establish the potential benefits of this diagnostic modality, allowing for optimal patient management and, therefore, improved outcomes. As described in this paper, there are changes regarding STE in patients with acromegaly. However, more data are needed to establish an acromegaly cardiomyopathy strain pattern, which could be useful in differentiating left ventricular hypertrophy, adding yet another disease to the differential list for the cardiologist. Endocrinologists can benefit from this imaging modality, too, as STE could also be a useful tool in monitoring treatment effects in this patient population. Cardiovascular outcomes drive survival rates, as shown by research; thus, a sensible marker of myocardial dysfunction, like STE, may guide clinical decision-making in the future.

## 7. Limitations

Six of the eleven discussed papers are part of the MAGYAR-Path study, which could be interpreted as selection bias. However, with such sparse available literature, no paper could be excluded on this basis. The conflicting results between different papers we discuss in this review may have resulted from highly heterogeneous study designs, which makes direct comparison difficult.

## Figures and Tables

**Table 1:** Summary of the analysed studies concerning speckle tracking echocardiography in patients with acromegaly.

Author, Year	Number of Patients with Acromegaly	Number of Patients in Control Group	Echocardiographic Strain Parameters	Strain Results
Popielarz-Grygalewicz, 2023 [6]	32	-	GLS	Impaired
Gadelha, 2022 [16]	25	44	GLS, radial strain, circumferential strain, and twist	No significant difference, except for radial strain, which was impaired
Koca, 2022 [19]	50	50	LV and LA strain	Impaired
Nemes, 2021 [23]	24	35	GLS, radial strain, circumferential strain, and twist	-
Uziębło-Życzkowska, 2020 [25]	30	30	GLS, LA strain	Impaired
Popielarz-Grygalewicz, 2020 [20]	43	52	GLS	Impaired
Kormányos, 2020 [22]	22	-	RA strain	Increased
Kormányos, 2020 [18]	19	21	GLS radial strain, and circumferential strain	Radial decreased
Kormányos, 2018 [21]	23	21	LA	-
Kormányos, 2018 [26]	20	18	Twist	Impaired
Volschan, 2017 [17]	37	48	GLS	No significant difference

LV—left ventricle; LA—left atrium; RA—right atrium; GLS—global longitudinal strain.

## Data Availability

The data can be shared upon request.

## Supplementary Materials

The following supporting information can be downloaded at https://www.mdpi.com/article/10.3390/life14111459/s1, Figure S1: Brain magnetic resonance images showing pituitary adenoma (A) and follow-up image after surgical removal (B).
