# Supplementary material for: Acromegaly in Speckle Tracking Echocardiography—A New Cardiac Hypertrophy Phenotype? Case Report and Review
Source: Life (Basel). 2024 Nov 11;14(11):1459. doi: 10.3390/life14111459 (PMC11595272; doi:10.3390/life14111459)
Supplement: Supplementary file 1 [file life-14-01459-s001.zip › life-3214468-Supplementary.pdf]

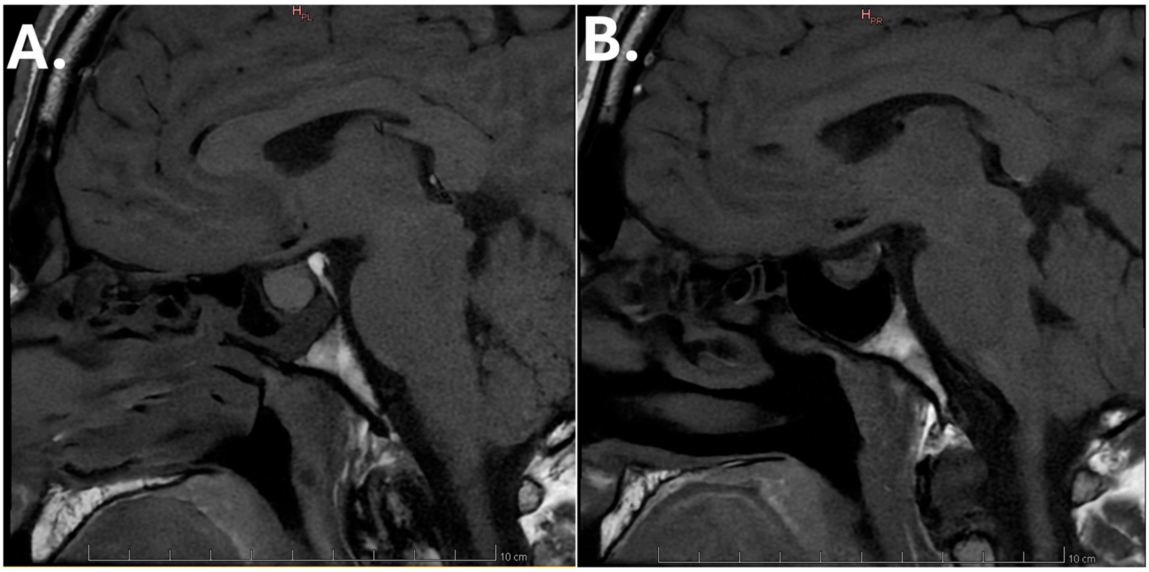


**Figure S1.** Brain magnetic resonance images showing pituitary adenoma. (A.) and follow-up image after surgical removal (B.).
